# An unusual outbreak of norovirus associated with a Halloween-themed swimming pool party in England, 2016

**DOI:** 10.2807/1560-7917.ES.2018.23.44.1700773

**Published:** 2018-11-01

**Authors:** Karthik Paranthaman, Ellen Pringle, Alison Burgess, Neil Macdonald, James Sedgwick

**Affiliations:** 1 Public Health England, National Infections Service, Field Service, London, United Kingdom; 2 Public Health England South East, Ashford, United Kingdom

**Keywords:** norovirus, outbreaks, United Kingdom

## Abstract

In October 2016, an outbreak of norovirus occurred among attendees of a Halloween-themed party at a public swimming pool in the south-east of England. Norovirus genogroup II was confirmed in 11 cases. In the retrospective cohort study of pool users, 68 individuals (37 female and 31 male), with a median age of 11 years (range: 0–50 years), met the case definition of developing diarrhoea or vomiting between 6 and 72 h after the pool visit. Multivariable analysis showed that increasing age was associated with a reduced risk of illness (odds ratio = 0.91; 95% confidence interval: 0.83–0.99). Pool behaviours (swallowing water) and the timing of visit (attending pool party after automatic dosing system was switched off) were independently associated with increased risk. Environmental investigations revealed that the automatic dosing system was switched off to reduce chlorine levels to an intended range of 0.5–1 parts per million to facilitate the use of a commercial red dye. There was a lack of compliance with the operator's own pool operating procedures, particularly on maintaining effective chlorine levels in pool water, recording of test results and recording of actions undertaken. This outbreak highlights the risks of lowering chlorine levels when using pool water colourants.

## Background

Norovirus is the most common cause of infectious intestinal disease in the United Kingdom (UK) with an estimated 3 million cases annually [1]. While outbreaks of norovirus commonly occur in healthcare and education settings, outbreaks associated with swimming pools are uncommon. It is thought that normally recommended standard chlorine levels in pool water, usually around 1 parts per million (ppm), provide effective disinfection against norovirus [2].

## Outbreak detection

On 31 October 2016, Public Health England (PHE) was informed by the manager of a local primary school in the south-east of England of a suspected outbreak of gastrointestinal illness among their schoolchildren. The school manager stated that the children had reportedly attended a Halloween-themed swimming pool party in a public leisure centre on 28 October.

It became apparent that a social media group had been set up by those who were affected by the outbreak and this led to the outbreak being widely covered in the national media before being reported to PHE. In line with standard procedures, PHE South East took the lead in investigating the outbreak with the support of other relevant stakeholder organisations. This paper summarises the key findings of the investigations.

### Setting

The leisure facility included an adult swimming pool, a toddler pool, a wave machine and flume. The swimming pool facilities are especially popular among families with young children during school holidays. The main pool, toddler pool and flume were linked to a common water system with an automatic chlorine dosing and filtration system.

Regular maintenance and monitoring of the pool water safety was done by staff of the facility, in accordance with guidelines laid out in a normal operating procedure (NOP) document, agreed and owned by the local authority. The NOP document was compliant with the UK national recommendations produced by the Pool Water Treatment Advisory Group (PWTAG) [3].

The automatic dosing system was in operation, which constantly monitored the free chlorine level and administered doses accordingly. The leisure facility was owned and operated by the local authority and regulated by the Health and Safety Executive (HSE).

A Halloween-themed pool party was held between 18:00 and 20:00 on 28 October, during which the pool water was dyed red using a commercial dye. The pool party was organised and advertised locally by the pool management as part of Halloween celebrations and was open to all members of the public. The event, held during school holidays, was aimed at families with young children. The user guide for the commercial dye product specified that pool water chlorine levels should be maintained below 1 ppm and that the colour would dissipate quickly if there was residual chlorine over 1 ppm. This requirement necessitated switching off the automatic dosing system that maintained chlorine levels in the pool water.

## Methods

### Microbiological investigations

Stool samples were sought from symptomatic cases who reported falling ill after attending the swimming pool. This convenience sample was drawn from children at the primary school and others who complained to the local authority about their illness. Samples were referred to the local PHE collaborating laboratory to test for the presence of bacterial (*Campylobacter* sp., *Salmonella* sp., Shiga toxin-producing *Escherichia coli* (STEC) and *Shigella* sp.), protozoal (*Cryptosporidium parvum,* *C. hominis and Giardia lamblia*) and viral pathogens (adenovirus, astrovirus, norovirus, rotavirus and sapovirus).

### Environmental investigations

Information on pool maintenance procedures and the pool party were obtained from the pool management. We also obtained the pool water records, which documented chlorine levels from the automatic dosing system (when in operation) and by manual testing (during the pool party) and actions undertaken by the pool operators before and after the outbreak. The pool management had implemented routine cleaning and superchlorination of the pool on the morning of 29 October to bring chlorine up to standard levels following the pool party. Following reports on 31 October of the potential outbreak linked to the pool, an additional round of superchlorination and deep cleaning of all communal areas was undertaken by the pool management on the same day. Therefore, environmental testing was not undertaken by PHE as part of the investigations. The pool management obtained water samples on 31 October for testing at their contracted accredited laboratory for standard indicator organisms (coliform bacteria, *E. coli* and *Pseudomonas aeruginosa*), and the results were subsequently shared with PHE.

### Epidemiological investigations

A retrospective cohort study was undertaken to capture information on pool users between 26 and 28 October. The pool water dye was not used on 26 and 27 October, before the Halloween-themed party on 28 October. A web-based survey questionnaire was prepared and piloted by PHE staff. It sought details on demographic variables, illness and potential exposures including pool visit time and the use of facilities such as changing rooms and toilets. Respondents were able to complete the survey on behalf of other pool users such as children but were asked to indicate if they had done so.

The survey questionnaire was communicated through a number of routes to pool users: through postal letters to those who complained to the Local Authority and those who submitted a stool sample, by posting a news item on the Local Authority’s website with a direct link to the survey site, and a cascade through the social media page set up by users affected by the outbreak. The survey was available for completion between 11 and 25 November (13–27 days after the party).

Cases were defined as individuals who visited the pool facility between 26 and 28 October 2016 and developed symptoms of diarrhoea and/or vomiting in the 6–72 h after their visit to the swimming pool. As the response rate was low, odds ratios (OR) were calculated as measures of association between demographic factors, exposures and illness. To investigate for presence of trend, we created two multicategorical variables from individual binary variables ordered from lower to higher risk: pool behaviours (did not enter pool, entered pool but did not submerge head, submerged head but did not swallow water, submerged head and swallowed water) and timing of visit (did not visit pool on 28 October, visited on 28 October before automatic dosing system was switched off, visited on 28 October after automatic dosing system was switched off but did not attend Halloween party, and attended Halloween party). Trend was determined by interpretation of OR across the levels of the multicategorical variable, in the presence of a statistically significant chi-squared test. Factors associated with illness in univariate analysis with a p value of < 0.2 and an OR > 1.0 were initially included in a multivariable model. Multivariable logistic regression analyses were conducted using a backwards stepwise approach, with age and sex included in the model as a priori factors. Confounding and effect modification were explored and also a dose–response relationship in terms of length of time spent in the pool.

## Results

### Microbiological investigations

Eleven children (median age: 9 years; range: 5–12 years) who attended the pool on 28 October and one adult who attended the pool on 27 October submitted stool samples. All 11 children tested positive for norovirus genogroup II. The 12 samples were negative for all other standard bacterial, protozoal and viral pathogens.

### Environmental investigations

Pool management reported that a risk assessment was done on the use of pool dye during a trial session in 2015, in which issues around supervising the pool with coloured water, required disinfection levels, and returning the pool back to normal with appropriate control measures were agreed. However, the procedural recommendations relating to the addition of dye were not formally recorded in the NOP document. Pool management reported that the dye had been used on at least 10 occasions since 2015 without any incidents or concerns, and that staff were aware of the need to maintain chlorine levels above 0.5 ppm to maintain effective disinfection.

The NOP specified that chlorine concentrations in the pool water should be tested and recorded a minimum of three times per day when the automatic dosing system was switched on and working, and every 2 h during manual dosing, i.e. when the automatic dosing system was switched off or not working. The NOP also stated that free chlorine levels were to be kept between 1.5 and 2.5 ppm to maintain effective disinfection and that levels should not fall below 0.5 ppm or rise above 4.0 ppm.

Pool water records showed that at 07:00 on 28 October, the level of free chlorine was adequate at 2.09 ppm. The automatic dosing system was turned off at 12:00 to allow the free chlorine level to drop below 1.0 ppm to allow the pool dye to work. The next recorded reading at 14:00 showed a free chlorine level of 0.24 ppm. No further recording of chlorine levels were made until the next morning at 07:00 when free chlorine was recorded as 0.0 ppm. Subsequently, a higher level of chlorination was implemented and this was confirmed by a free chlorine level of 2.9 ppm at 10:00 on 29 October.

Pool management reported that the automatic dosing system was turned back on for at least 1.5 h at 14:00 when free chlorine level dropped below 0.5 ppm and that free chlorine levels were monitored on 28 October but these actions and monitored chlorine levels were not entered in the pool water records. They also reported that a failure of computer systems on 28 October caused significant disruption to the operation of the site but that the automatic dosing system was functioning correctly on that day.

No reports of faecal contamination or vomiting episodes at the pool facilities were made to the leisure centre staff on 28 October. Two staff working by the poolside as lifeguards on 28 October reported developing symptoms of diarrhoeal illness in the 48 h following their shift. They did not report entering the pool but would have been involved as lifeguards for the pool and cleaning the toilets and changing rooms throughout their shift.

Routine cleaning and disinfection of the pool facilities was undertaken on 29 October. Upon notification of the outbreak, an additional round of super-chlorination of the pool and deep cleaning of the communal areas was undertaken on 31 October. Water samples from the pool taken on 31 October confirmed the absence of standard bacterial pathogens (coliform bacteria, *E.coli* and *P. aeruginosa*).

### Epidemiological investigations

A total of 93 valid questionnaire responses were included in the analysis. As the number of pool users between 26 and 28 October was not recorded due to a failure of computer systems, the response rate for the cohort study could not be calculated. Based on staff reports and sale of entry tickets, the pool management estimated that ca 150 people had used the pool facilities on the days between 26 and 28 October and an additional 140 persons had attended the Halloween party between 18:00 and 20:00 on 28 October.

Among the 93 respondents, 68 (73%) met the outbreak case definition of developing diarrhoea or vomiting between 6 and 72 h after the pool visit. Seven respondents reported symptom onset more than 72 h after the pool visit (range: 82–154 h); we therefore assumed that they represented either secondary transmission or an unrelated illness and included them as non-cases. One respondent who reported symptoms within 3 h of visiting the pool on 27 October was also classified as a non-case.

Children (up to 16 years of age) were more likely to be ill compared with adults (52/53 vs 16/40; p < 0.001, chi-squared test). The age range for all respondents was 0–57 years; the median age was 11 years for cases and 39 years for non-cases (p < 0.001 by Wilcoxon rank-sum (Mann–Whitney) test for age). Among the cases, 37 were female and 31 were male.

**Figure 1:**
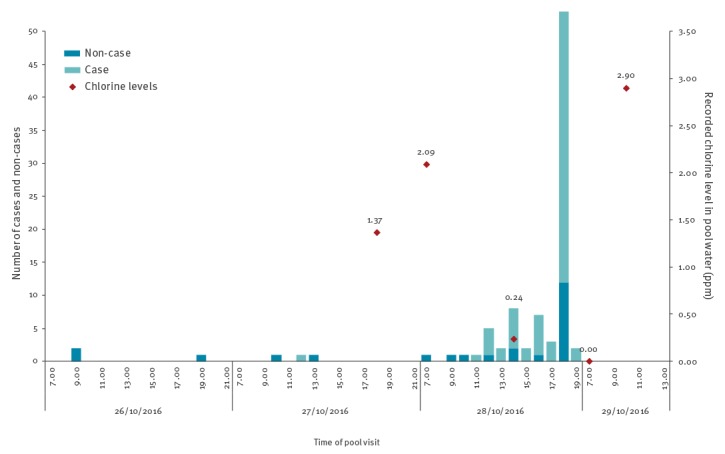
Time of pool visit for cases and non-cases against recorded chlorine levels, norovirus swimming pool outbreak, England, October 2016 (n = 92^a^)

^a^ Pool visit time was not available for one respondent.

The majority of respondents (86/93) had visited the pool on 28 October, and this included one respondent who reported attending on all 3 days between 26 and 28 October. Three and four respondents reported attending the pool on 26 and 27 October, respectively. Except for one case who reported attending the pool at 12:00 on 27 October, all other cases reported attending the pool on 28 October. The time of pool visit for cases and non-cases is shown in Figure 1.

**Figure 2:**
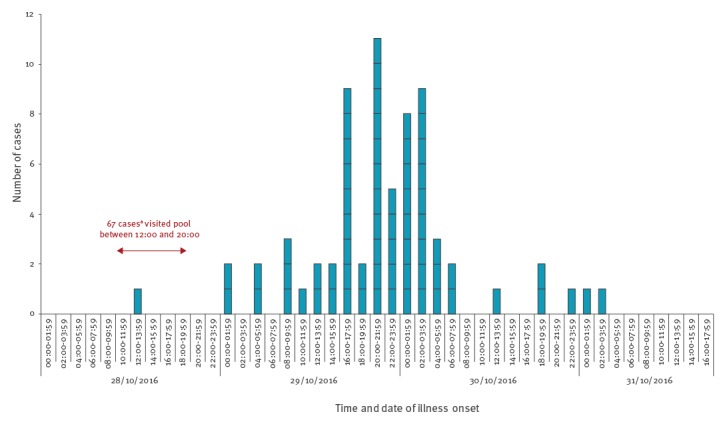
Time of onset of illness for cases, norovirus swimming pool outbreak, England, October 2016 (n = 68)

^a^ One case visited the pool on 27 October at 11:00 h.

Symptoms reported by the cases were typical of norovirus infection, with vomiting (65/68), cramps (63/68) and nausea (54/68) being the most frequent. Symptom onset peaked ca 24 h after the end of the Halloween party, with a median incubation period in cases of 28 h (range: 6–56 h) from the time of visit (Figure 2). The median length of symptoms was 3 days (range: 1–6 days). Most cases (n = 25) had a duration of symptoms of 2 days, but 21 cases reported symptom duration of 4 days or longer. Regarding the use of healthcare services, six cases sought advice from NHS 111 (a freephone advice and referral service for health issues), seven from their general practitioner, four attended the Accident and Emergency Department (A and E) and two were admitted to hospital for treatment. Overall, the 61 cases reported missing, collectively, at least 125 days of work or school as a result of their illness.

Among the 93 respondents, 85 reported entering the swimming pool, of whom 67 became cases. Among the remaining eight who did not enter the pool waters, there was one case and seven non-cases. Of the 93 respondents, 18 (21%) reported showering before entering the pool and 68% (56/93) after leaving the pool. A total of 35 respondents reported using a toilet during their visit. Fifty-nine respondents reported attending the Halloween party, of whom 46 became symptomatic and were classified as cases.

All cases visited the pool after 12:00 on 28 October except for two cases: one entered the pool at 12:00 on 27 October and became symptomatic 24 h later, and the other reported entering the pool at 11:00 on 28 October and spent more than 120 min in the pool. In the univariate analysis (Table 1), visiting the pool on 28 October was associated with an OR of 21.2 (95% confidence interval (CI): 2.26–986 p < 0.001). In the multicategorical variables, a statistically significant trend was detected for both pool behaviours and timing of visit: Swallowing water (OR = 203; 95% CI: 16.3–2,536 compared with not entering the pool, and attending Halloween party (OR = 23; 95% CI: 2.52–209) compared with not visiting on 28 October, were associated with higher odds of illness. The odds of being a case decreased statistically significantly with each year of increase in age (OR = 0.87; 95% CI: 0.83–0.92; p < 0.001) but were not associated with sex (OR = 2.65; 95% CI: 0.87–9.06; p = 0.059).

**Table 1:** Univariate analysis, norovirus swimming pool outbreak, England, October 2016 (n = 93; cases = 68)

Exposure		Exposed cases	Exposed non-cases	OR	95% CI	p value
Visited pool on 26 October^a^		0	4	0.06	0.00–0.52	0.001
Visited pool on 27 October^a^		1	4	0.08	0.00–0.88	0.006
Visited pool on 28 October^a^		67	19	21.2	2.26–986	< 0.001
Used any changing room		67	18	26.1	2.92–1190	< 0.001
Showered before entering pool		15	3	2.08	0.51–12.2	0.28
Showered after using pool		42	14	1.27	0.45–2.54	0.62
Used any toilet		24	11	0.69	0.25–1.98	0.44
Pool behaviours	Did not enter pool	Reference				< 0.001
	Entered pool but did not submerge head	1	11	0.63	0.03–11.9	
	Submerged head but did not swallow water	8	5	11.2	1.04–120	
	Submerged head and swallowed water	58	2	203	16.25–2,536	
Timing of visit	Did not visit on 28 October	Reference				< 0.001
	Visited on 28 October before 12:00 (chlorine auto-doser switched on)	0	3	0	Not estimable	
	Visited on 28 October after 12:00 (chlorine auto-doser switched off) but did not attend Halloween party	21	4	31.5	2.93–337	
	Visited on 28 October and attended Halloween party between 18:00 and 20:00 (chlorine auto-doser switched off)	46	12	23	2.52–209	

CI: confidence interval; OR: odds ratio.

^a^ One respondent attended on all three days.

The multivariate model (Table 2) was based on 91 observations because information on age was missing for two respondents. The initial model included age, sex, changing room use, pool behaviours and timing of visit. Age was included as a linear function after confirmation that quadratic and cubic functions did not provide significantly better model fit. The use of changing room cofounded the effect and was therefore retained in the final model. After adjustment, increasing age, pool behaviours and timing of visit were all independently associated with illness. The 95% CIs for individual factors in the two multicategorical variables were very wide; nevertheless, the point estimates (ORs) demonstrated a statistically significant trend of increasing risk from factors with lower to higher risk.

**Table 2:** Results of the multivariate analysis, norovirus swimming pool outbreak, October 2016, England (n = 91^a^)

Exposure		Adjusted OR	95% CI	p value
Age (continuous)		0.91	0.83–0.99	0.0142
Pool behaviours	Did not enter pool	Reference		< 0.001
	Entered pool but did not submerge head	0.02	Not estimable	
	Submerged head but did not swallow water	1.15	Not estimable	
	Submerged head and swallowed water	12.5	Not estimable	
Timing of visit	Did not visit on 28 October	Reference		0.0074
	Visited on 28 October before 12:00 (chlorine auto-doser switched on)	0	Not estimable	
	Visited on 28 October after 12:00 (chlorine auto-doser switched off) but did not attend Halloween party	9.99	0.28–351	
	Visited on 28 October and attended Halloween party between 18:00 and 20:00 (chlorine auto-doser switched off)	36.1	0.95–1362	
Used changing room		6.55	Not estimable	0.71

CI: confidence interval; OR: odds ratio.

^a^ Two individuals did not provide age.

Interaction between the timing of visit and pool behaviours was tested for but was not significant. A dose–response effect, based on the length of time individuals spent in the swimming pool, was not observed as the majority of individuals reported spending 60–120 min in the pool.

Some respondents commented on a sub-optimal state of cleanliness at the pool and in changing room and toilets, but none reported witnessing anyone being unwell in or near the pool water.

## Discussion

Epidemiological and microbiological evidence confirmed that an outbreak of norovirus occurred among users of the swimming pool facilities on 28 October 2016. Findings from the retrospective cohort study showed that those who attended the pool after the automatic dosing system was switched off on 28 October, or submerged their head in pool water or swallowed water, had a higher risk of becoming ill compared with those who did not. Furthermore, the outbreak ended abruptly when chlorine levels were returned to standard levels the following day. An internal investigation by the local authority confirmed that free chlorine levels in the pool water were recorded as between 0.5 and 1 ppm on all previous occasions when the dye had been used. This suggests that this outbreak was most likely linked to reduced chlorine levels (below 0.5 ppm) in pool water on 28 October. Environmental evidence showed a lack of compliance with the pool management’s own operating procedures, particularly on maintaining effective chlorine levels in pool water, recording of routine test results and recording of actions undertaken.

Norovirus is commonly transmitted via direct contact with infected persons (person-to-person), consumption of contaminated food and water or contact with contaminated environment. Given the typical point-source outbreak curve (Figure 1), person-to-person transmission was not likely to be the primary cause of transmission in this outbreak. Similarly, acquisition of norovirus through contamination of food is not likely as food was not served to the attendees of the Halloween pool party on 28 October.

To the best of our knowledge, there have been no published reports of infectious disease outbreaks or adverse health effects linked to the use of colouring dye in swimming pools in England or elsewhere. The technical data sheet for the product stated that the preparation contained no substance classed as hazardous per directive 67/548/EEC but did not list the composition or ingredients. The colouring dye was manufactured in Country X and the company website listed distribution centres in several European and non-European countries across the world. Since the colouring dye required chlorine levels to be maintained below 1 ppm, maintaining effective chlorine levels and the colour effect at the same time can be very challenging to achieve, especially in a large pool facility. On balance, we considered that any health effects from the use of the dye were likely to arise from the temporary reduction of chlorine levels rather than due to the dye itself, although there is no evidence to support or refute this assumption. 

While a specific contamination event could not be identified, epidemiological evidence is consistent with a point source outbreak. It is well known that in outbreaks of norovirus associated with recreational water facilities, a contamination event (faecal or vomiting contamination) at the pool may not always be identified [2,4]. 

Norovirus is the most common pathogen causing gastrointestinal illness in England [1]. It is known that up to 32% of norovirus infections may be asymptomatic and that a gram of faeces from an infected person can contain ca 100 billion viral copies [5,6]. Noroviruses can be found in faeces in variable numbers even before the onset of symptoms in infected individuals. On average, people have ca 0.14 g of faeces on their bodies when they enter a swimming pool [7]. For children, this amount can vary from 0.01 to 10 g [8]. The minimum infectious dose for norovirus infection may be as low as 6–10 virus particles, therefore contaminated water poses a significant public health risk [9]. The most likely explanation for the outbreak reported here is that a significant contamination incident by an asymptomatic or symptomatic pool user (unreported to pool management) introduced a large amount of norovirus particles in the pool water system and the reduced level of free chlorine (below 1 ppm) on this particular date was inadequate to effectively disinfect the entire body of contaminated pool water.

Scientific evidence on chlorine levels in swimming pool waters and transmission of norovirus is limited. In a review of recreational waterborne outbreaks, the authors concluded that adequate chlorination can be an important component in the prevention of norovirus outbreaks originating from swimming pools [10]. Outbreaks of norovirus in swimming pools are infrequent, suggesting that normally recommended chlorine levels in swimming pool waters may be adequate to limit transmission [2]. Guidance from the United States Centres for Disease Control and Prevention (CDC) states that 99.9% of noroviruses are inactivated at a chlorine concentration of 1 mg/L (=1 ppm) with exposure of 4.2 s [11,12]. The CDC recommend a free chlorine concentration of at least 1 ppm in pools [13]. In contrast, UK guidance from the PWTAG states that free chlorine levels *should be maintained at 1 mg/L or below, to an absolute minimum of 0.5 mg/L* [3]. Because of a lack of published studies, it is unclear if free chlorine levels between 0.5 and 1 ppm would be effective in preventing transmission of norovirus.

In this outbreak, children were disproportionately affected compared with adults. This finding is line with scientific consensus that children are at higher risk of getting infected in waterborne recreational outbreaks; this is due to their naïve immune systems and behavioural factors such as poor hand-washing practices and increased likelihood of swallowing water [14,15].

Showering before the use of swimming pools is recommended to minimise the risk of biological and chemical contamination of the pool by users [16]. Similarly, showering after the use of a pool is recommended to remove any body surface contamination by biological and chemical agents in the pool water. In this outbreak, only 19% of respondents reported using the shower before and 60% after use of pool. This low level of compliance suggests poor awareness among users of the preventative role of showering in maintaining pool hygiene and in reducing the risk of illness. Interventions to improve the use of showers before and after use of pool by customers should be considered.

There are some limitations to this study. Firstly, the response rate of the survey was low, at ca 35% for the party attendees, and there were few responses from non-cases. Nevertheless, we were still able to detect some associations between pool exposures and illness. Secondly, the majority of respondents were parents completing the survey on behalf of their children, most of whom were unwell, which may have affected the accuracy of reported exposures such as swallowing water. Thirdly, the actual level of chlorine in the pool water after 14:00 on 28 October is unknown. Finally, we did not undertake an independent review of pool maintenance procedures.

Despite the cause being norovirus, the outbreak had a moderate impact in terms of the numbers who became ill, those who sought medical care and missed school or work activities as a consequence of their illness. This outbreak highlights the need for maintaining effective chlorine levels to mitigate the risk of norovirus transmission in swimming pools, and in particular the need for additional vigilance during periods when automatic dosing systems are switched off. Swimming pool operators need to be aware of the health risks when using colouring agents that require lowering of normally recommended chlorine levels.
